# Equestrian-related maxillofacial injuries—a five-year retrospective review

**DOI:** 10.1007/s11845-025-03995-4

**Published:** 2025-07-12

**Authors:** Brian Maloney, Min Seo Jung, Gerard Kearns, Conor Bowe

**Affiliations:** National Maxillofacial Surgery Unit, St. James’s Hospital, Dublin, Ireland

**Keywords:** Equine, Facial fracture, Horse-related, Maxillofacial injury, Trauma

## Abstract

**Background:**

Equestrian-related activities inherently involve the risk of serious injury. There are few reports of maxillofacial injury patterns and management arising from equestrian activities in the literature.

**Aims:**

To investigate maxillofacial fracture presentations at a tertiary trauma centre to identify injury profiles of patients who have sustained equine-based injuries over 5 years.

**Methods:**

This work involved a retrospective review of all trauma presentations to our unit over 5 years. The data collected included patient demographics, injury mechanisms and patterns, and management of fractures.

**Results:**

The study identified 73 patients with facial fractures resulting from activity with horses between 2020 and 2025. A total of facial fractures were recorded. The mean age was 39.5 (11–86) years. There were 40 (55%) males and 33 (45%) females. Mounted riders accounted for 41% of cases, with 59% being unmounted. The incidence of head injury was 5%. A further 5% had an associated spinal injury. The most common specific mechanism of maxillofacial injury was a direct kick, in 55% of cases. The most commonly affected facial region was the middle third (81%), with zygomaticomaxillary complex fractures most commonly recorded (39%). There was a statistically significant increase in the risk of head injury associated with frontal bone fracture in this group.

**Conclusion:**

Activities involving horses pose a high risk of maxillofacial injury. Education should be promoted to increase the usage of helmets with a particular design to protect the facial skeleton, whether mounted or unmounted.

## Introduction

Ireland has a rich equestrian heritage, with horses playing a significant role in culture, history, and the economy. Horse riding is a popular pastime primarily practised in a recreational setting. The spectrum of equestrian activities is broad and ranges from agriculture to competitive sport (Zuckerman et al., 2015). It is estimated that over 50,000 people are regularly involved with sports horses in Ireland every week, equating to almost 1% of the total population [1].

Equestrian activities involve the risk of serious injury, and competitive equestrian sports, in particular, are dangerous, with one of the highest mortality rates of all sports [2]. An estimated one in five individuals with involvement in horse-related activities in either a recreational, occupational, or professional capacity will suffer an equine-related injury (ERI) necessitating hospitalisation [3]. Horse-related trauma constitutes a sizeable proportion of presentations to emergency departments and referrals to tertiary trauma centres worldwide, accounting for approximately 24 in 100,000 presentations and 0.17 in 100,000 deaths [4].

Equestrian injuries have a diverse injury presentation owing to the variable mechanisms involved. While the overall incidence of trauma is lower than in other sports, there is a disproportionately higher rate of serious injury. An adult horse can weigh approximately 500 kg, travel up to 65 km/h, and has the potential to deliver a kick with over 10,000 N of force [5]. Horse-related injuries can occur while the rider is mounted or unmounted (usually due to being crushed, trodden on, or kicked). Mounted riders are at risk of high-speed ejection from the animal due to minimal restraint. These risks are compounded by the unpredictable nature of horses and the often lack of compliance with protective equipment by the handler, in particular when the handler is unmounted, for example, working in the stable with horses. There is also the additional risk of being struck after the fall and being trampled upon.

Therefore, equestrian activities are considered dangerous, and the injuries sustained can be more severe than those encountered in other sporting activities. Horse-related trauma has been extensively researched [4, 6–12]. However, maxillofacial trauma arising from equine activities is often overlooked. According to Norwood et al., the head and craniofacial region are the most likely body regions to be injured [13]. The facial skeleton is vulnerable to injury, as conventional protective headgear only protects the cranial portion of the skull. A review of the literature reveals limited studies that exclusively focus on equestrian-related maxillofacial injuries [14–17].

The objective of this study is to analyse injury patterns of equestrian-related maxillofacial trauma presentations at a tertiary trauma centre in Ireland and to clarify injury profiles of patients who have sustained equine-based injuries over 5 years.

## Methods

A retrospective review was carried out to identify equine-related maxillofacial injuries presenting to a tertiary trauma centre (National Maxillofacial Surgery Unit, St. James’s Hospital) over 5 years, between March 2020 and March 2025. Data was gathered from a computerised trauma database and contemporaneously maintained logbooks of all trauma presentations [18].

All patients who sustained horse-related maxillofacial injuries referred to the National Maxillofacial Unit at St. James’s Hospital were included in this study. Demographic data collected included age, gender, date of accident, mechanism of injury, maxillofacial injury (hard and soft tissue), other non-maxillofacial injuries, head trauma, and management.

The mechanism of injury was classified as follows: mounted and unmounted. The activity at the time of injury was also recorded as follows: mounted (horse racing, hunting, show jumping, recreational hacking, other) and unmounted (table activities, leading to the field, leading into horse transport).

The collated data was analysed using descriptive and analytic statistics using the Statistical Package for the Social Sciences version 16 (SPSS Inc., Chicago, IL, USA). Bivariate associations were tested for statistical significance using chi-square tests, and values of less than 0.05 were considered significant.

## Results

The study identified 73 patients with facial fractures resulting from activity with horses over the 5 years between 2020 and 2025. A total of 98 injuries and 83 facial fractures were recorded in this patient cohort (Table 1). The mean age of presentation was 39.5 (11–86) years. There were 40 (55%) males and 33 (45%) females. Male predominance was observed across all age groups. Fractures were more commonly seen in individuals under 40 years of age (55%).

**Table 1:** Frequency and pattern of maxillofacial and non-maxillofacial injuries, need for admission (surgery), and compliance with headgear usage

Injury pattern	Male (*n*)	Female (*n*)	Total (*n*)	Total (%)	*P* value
ZMC	21	11	32	39%	0.100
Orbit	8	7	15	18%	0.898
Mandible	7	5	12	14%	0.318
Nasal bone	6	3	9	11%	0.445
NOE	2	4	6	7%	0.270
Frontal	3	0	3	4%	0.108
Supraorbital rim	1	0	1	1%	0.360
Le fort	3	2	5	6%	0.809
Dentoalveolar	2	1	3	-	0.673
Soft tissue	6	6	12	-	-
Total injuries (*n*)	59	39	98		
Facial fractures (*n*)	51	32	83		
Admission	17	14	31	42%	
Single fracture	29	24	53	72%	
Multiple fractures	12	8	20	28%	
Spinal injuries	2	2	4	5%	
Head injury	2	2	4	5%	
Helmet compliance	-	-	21/60	35%	
Mounted	-	-	18/29	62%	
Unmounted	-	-	3/31	10%	

During the study period, 5321 patients presenting with facial fractures to our unit were recorded between March 2020 and 2025. Equestrian-related fractures (*n* = 73) represented 1% of the total number of patients with facial fractures over this timeframe.

### Type of fracture

**Fig. 1:**
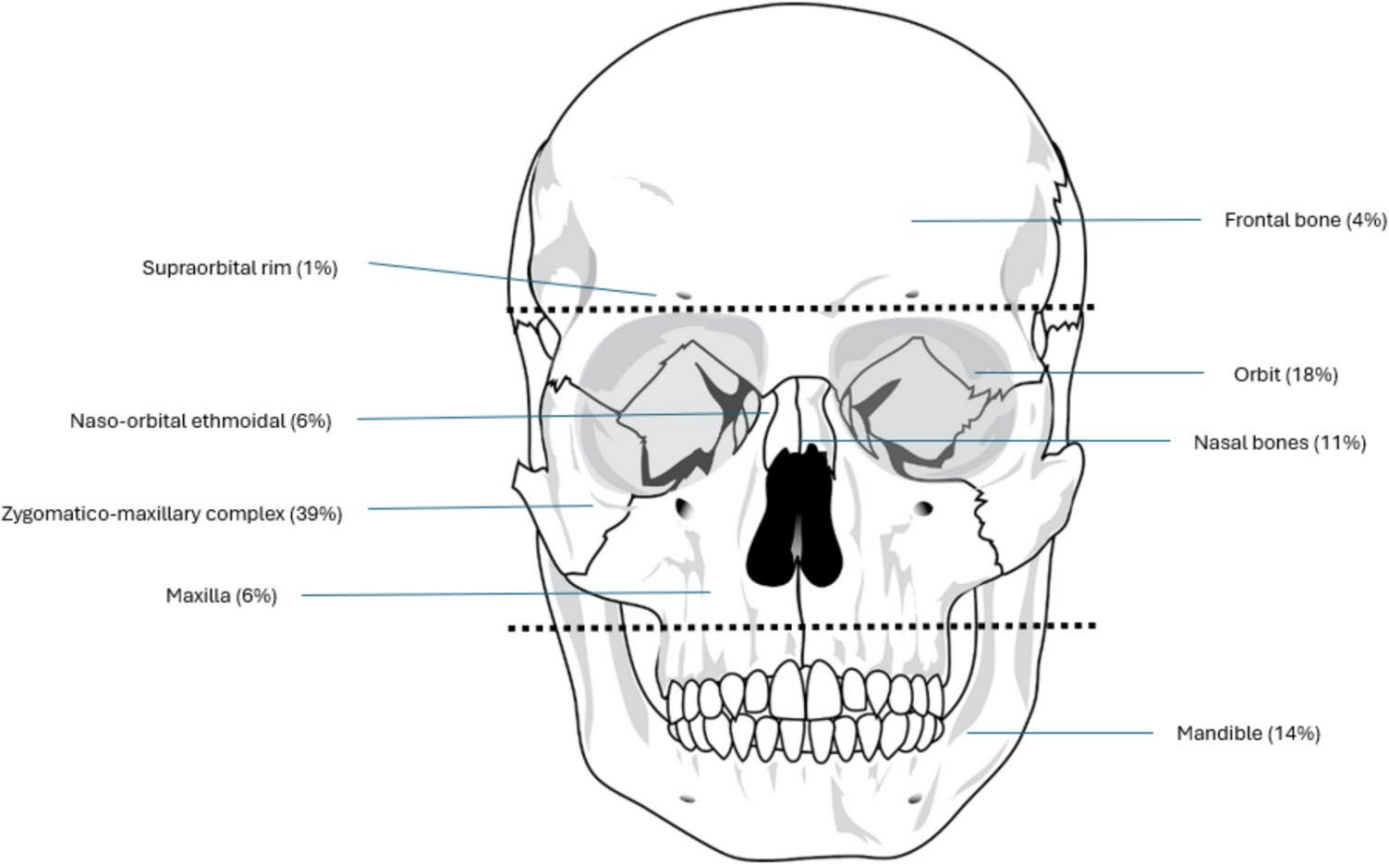
Distribution of facial fractures by facial thirds (upper, middle and lower) as indicated by dashed lines related to equine injuries (Source: Pexels.com)

Patients were grouped according to the third of the facial skeleton in which the fracture(s) occurred. The facial skeleton was divided into upper, middle, and lower thirds (Fig. 1). The most commonly affected region was the middle third, which accounted for 81% of fractures (Table 1). Within the middle one third, the fracture pattern was as follows: zygomaticomaxillary complex (39%, *n* = 32), isolated orbit (18%, *n* = 15), naso-orbital ethmoidal (7%, *n* = 6), nasal bone (11%, *n* = 9) and Le Fort pattern fractures (6%, *n* = 5). The upper third accounted for 5% (*n* = 5) of fractures, resulting from three frontal bone and one supraorbital rim fracture. The lower third was associated with the remaining 14% of facial fractures, all of which involved the mandible.

Multiple facial fractures were observed in 28% of cases (Table 1). The inclusion of dentoalveolar and soft tissue lacerations increased the total number of injuries to 98. Soft tissue lacerations were observed in 16% (*n* = 12) of cases. Three people (4%) suffered concurrent dentoalveolar trauma.

**Fig. 2:**
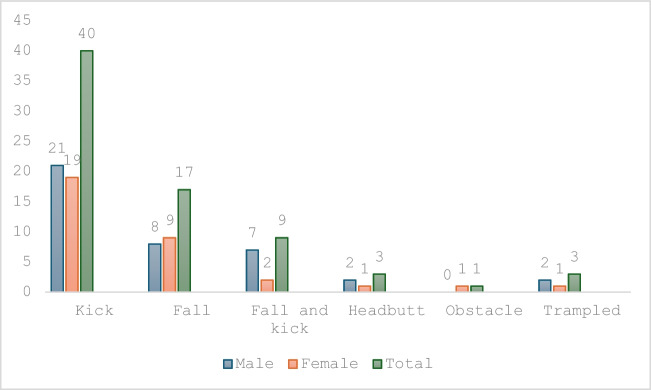
Proportion of injuries caused by each mechanism

The majority of fractures occurred in males, representing 71% of all facial fractures (*n* = 59) (Fig. 2). Males were also more likely to sustain all types of facial fractures, except naso-orbital ethmoidal fractures, the majority of which (67%, *n* = 4) affected females (Table 1).

### Mechanism of injury (Table 2)

**Table 2:** Association between mechanism and pattern of maxillofacial fracture

		*Mechanism of injury*						
		Kick	Fall	Fall and kick	Headbutt	Trampled	Obstacle	*p* value
*Pattern of fracture*	ZMC	15	7	7	0	1	2	0.102
	Le Fort	4	1	0	0	0	0	0.873
	Mandible	8	2	0	2	0	0	0.121
	Nasal bones	6	2	1	0	0	0	0.875
	Dentoalveolar	3	0	0	0	0	0	0.764
	Orbit	9	5	1	0	0	0	0.667
	NOE	4	1	0	1	0	0	0.555
	Frontal	2	0	1	0	0	1	0.118
	SOR	0	0	1	0	0	0	0.205

The mechanism and aetiology of injury were divided into two broad groups: mounted (on horseback) and unmounted (stable duties, grooming, loading into vehicle, feeding, etc.). The majority of injuries occurred in the unmounted group, 59% (*n* = 53), when compared with the mounted group, 41% (*n* = 31).

Falls from a mounted position were responsible for 17 (23%) of injuries. The remaining cases were associated with falls and subsequent kicks (12%), headbutts (4%), trampling (4%), and riding into an obstacle (1%). Zygomatico-maxillary complex fractures were the most common injury from a mounted position (*n* = 17) (Figs. 1 and 2).

The most common specific mechanism of maxillofacial injury was a direct kick, in 55% (*n* = 40) of cases, resulting in the following injury pattern: zygomatico-maxillary complex (*n* = 15), mandible (*n* = 8), and isolated orbital fractures (*n* = 9) (Table 2). Furthermore, 80% (*n* = 4) of Le Fort midface fractures resulted from a kick from an unmounted position.

The specific activity at the time of injury was also assessed (Table 3). Information was available from 60 of 73 patients from the study population who responded to a follow-up questionnaire. The results of this aspect of the study are outlined in Table 3. Thirty-one of 60 patient injuries (50%) were sustained when unmounted. Of note, 14 of 31 (45%) occurred when leading the horse, and 12 of 31 (39%) occurred in the stable when grooming, tacking, and feeding. A further 4 (13%) were sustained linked to stable vet or farrier involvement with the horse, confirming a majority of unmounted injuries occurring in the stable. The injuries seen in those on horseback (mounted), *n* = 29, were mainly related to amateur show jumping or arena activities, *n* = 12 (40%).

**Table 3:** Distribution of specific activities resulting in injury

Activity	*N*
*Mounted (on horse)*	*n* = 29
• Show jumping/arena (amateur)	*n* = 12
• Hacking out	*n* = 11
• Hunting	*n* = 2
• Professional jockey (national hunt)	*n* = 2
• Carriage driving	*n* = 2
*Unmounted (off horse)*	*n* = 31
• Loading/leading	*n* = 15
• Stable activities	*n* = 16
o (Tacking/grooming/feeding)	*n* = 12
o (Farrier/vet)	*n* = 4

The use of helmets was recorded in both groups, with 18/29 (62%) wearing helmets in the mounted group against 3/31 (10%) in the unmounted group (Table 1). As might be expected, fewer patients used helmets in the unmounted (non-riding) group.

### Management

**Fig. 3:**
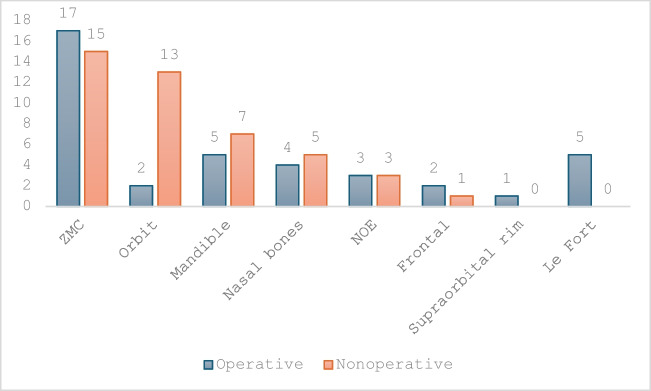
Management of fracture type

Regarding those patients in the overall study population with facial fractures, 31 of 73 patients (42%) were admitted for surgical management of their injuries (Table 4). The remaining 42 patients (58%) were treated non-operatively. Of the 83 facial fractures in total, 39 (47%) required operative management under general anaesthesia (Fig. 3). Specifically reviewing the management of variable injury patterns, 17 patients (53%) with ZMC fractures required open reduction and internal fixation (ORIF). Closed reduction was carried out for mandible fractures in 42% of cases, all of which involved isolated mandibular condyle fractures, with the remaining mandibular fractures, 58%, treated with ORIF. All individuals with Le Fort pattern midface fractures (*n* = 5) required operative intervention and ORIF. The majority of isolated orbital fractures were treated non-surgically, 13 patients (87%). The most common injury pattern requiring surgical intervention was a zygomaticomaxillary complex fracture from a kick (*n* = 9) (Table 4).

**Table 4:** Operative vs conservative management per injury pattern

Injury	Operative	Conservative	%	*p* value
ZMC	17	15	53%	0.104
Orbit	2	13	13%	*0.01*
Mandible	5	7	42%	0.951
Nasal bones	4	5	44%	0.898
NOE	3	3	50%	0.697
Frontal	2	1	67%	0.386
SOR	1	0	100%	0.241
Le Fort	5	0	100%	*0.007*
Dentoalveolar	2	1	67%	0.386

The hospitalisation rate was higher in patients under 40 years of age (*n* = 17, 43%) compared with those over 40 years (*n* = 13, 39%). There was no difference in admission rates between males and females (Table 1).

### Other injuries

In this patient cohort, concurrent head injuries were diagnosed in 5% of cases, four in males and one in a female patient (Table 1). Neurosurgical input was non-operative in all cases of head injury. One patient suffered a base of skull fracture with extension into the anterior cranial fossa with an associated subarachnoid haemorrhage. Three cases were associated with a direct kick, one due to a fall and kick, and one due to trampling. No patient died as a result of their intracranial injuries.

Eleven patients (15%) had injuries in more than one body region, excluding head injuries (Table 1). The most commonly injured area was the spine (5%, *n* = 4), followed by the abdomen (4%, *n* = 3). The lumbar spine was the most common location for fracture amongst all spinal injuries. The remaining injuries involved fractures in the upper (3%, *n* = 2) and lower (3%, *n* = 2) extremities.

### Statistical analysis

No statistically significant associations were identified between the mechanism of injury and the pattern of maxillofacial injury (Table 2). Le Fort pattern fractures had a significant likelihood of requiring operative management (0.007). In contrast, isolated orbit fractures were more likely to be treated non-operatively (0.01). The requirement for surgery was equal for both males and females (0.995), with no significant differences in the incidence or type of maxillofacial fracture between the two genders.

Discussing head injury, there was a statistically significant risk associated with frontal bone fractures (0.03). There was no association between the mechanism of injury and head injury (0.256). Also, falls from a mounted position demonstrated an increased likelihood of spinal trauma compared to other mechanisms (0.029).

## Discussion

The equestrian industry in Ireland provides a significant contribution to the economy. According to a report by Horse Racing Ireland, the Irish sport horse industry is worth approximately €816 million annually, employing approximately 14,000 individuals, within the competitive, recreational, and breeding sectors [1]. Those involved in direct animal contact are at risk of equestrian-based injury. Trauma arising from horse-related activities has been shown to have a high financial burden on healthcare systems, surpassing many other popular sports [11].

This is the first study addressing equine-related maxillofacial fractures in Ireland. The work aimed to capture and characterise the epidemiology, aetiology, and management of horse-related maxillofacial fractures referred to a tertiary maxillofacial unit over 5 years. Patients who were managed non-operatively were also included in the study population. The injuries ranged from relatively minor, such as lacerations or dental injuries, to fractures of the facial skeleton, with varying degrees of severity.

Equestrian-related facial injuries constitute a small proportion of the totality of maxillofacial injury presentations at the Maxillofacial Unit, as illustrated when reviewing the total number of maxillofacial injuries during the study period. While horse-related fractures account for a small proportion of patients with facial trauma (1%), 42% of this cohort required surgery. This is in contrast to all other sporting injuries, of which only 11% required admission in our unit. This finding supports the existing literature that shows a disproportionate number of serious injuries related to equestrian activities and highlights that equestrian sports may be amongst the most dangerous of all recreational sports [12, 14]. The velocity and mass of horses result in equine-related injuries being compared to road traffic accidents [13]. It is therefore not surprising that equestrian-related facial injuries predispose to more unfavourable fractures, requiring a higher proportion of surgical intervention and hospital admissions.

A young, mainly female population has been previously identified as the most at-risk group for equine-associated trauma [17, 19]. The reasons suggested for this are that there are often a higher number of young female participants involved in equestrian sport [20]. This was not supported in this study, wherein males were overrepresented. The proportion of males in equestrian sport is occasionally reported to be higher; therefore, this finding is consistent with the specific demographics of the present study [21]. Most individuals in this study were young, with 55% under the age of 40. Men tended to be involved in equestrian activities at an older age than their female counterparts. The statistical analysis did not demonstrate any differences between males and females relating to the need for surgery or the pattern of injury that was sustained.

In this study, mid-third facial fractures were the most common pattern, similar to previous reports [15, 22]. Zygomaticomaxillary complex fractures were the most frequently reported pattern overall, followed by isolated orbital and mandible fractures. This is in keeping with previous limited studies [16, 17, 22]. More than half of individuals with fractures in this report were suitable for non-operative management (53%).

A total of 28% of presentations involved multiple facial fractures, which introduces the risk of more subtle but perhaps more serious injuries being overlooked because of a more obvious or distracting injury. Kicks were most likely to result in multiple facial injuries, followed by falls. Therefore, patients suffering an equine-related facial injury must receive assessment in accordance with Advanced Trauma Life Support (ATLS) guidelines.

Horseback riding is a common recreational activity that can lead to injuries to both mounted and unmounted participants. Several studies have identified falls as the primary aetiological factor [9, 14, 23]. However, it has also been reported that, although most injuries occur during recreational riding, approximately 15% of injuries occur in non-riding activities, such as feeding, handling, shoeing, and saddling [23]. In the case of maxillofacial injuries in particular, there is a reported increase in injuries when riders are unmounted [15–17, 22]. These reports are supported by the findings of the present study, where a large number of injuries were sustained while unmounted. The distribution ratio of fractures in mounted versus unmounted was 1:1.6. This may reflect the demographics of presentations, in that many injuries were occupational.

Looking specifically at the activities that resulted in maxillofacial injuries, a supplementary questionnaire answered by 60 of the original 73 study patients revealed that 31 patients sustained injuries while unmounted. The majority of this group (*n* = 16) were involved in stable activities (grooming, tacking, feeding, farrier, vet), and the remaining 15 patients were leading or loading horses. Only three of these 31 (10%) patients were wearing helmets at the time of injury. This underlines the often underestimated risk to those handling and working with horses in stables, and it is advocated that those handling horses in this environment should wear helmets. It may be argued that the use of helmets may not prevent facial injuries; however, it may mitigate against the risk of more serious head injuries. The remaining 29 patients responding to the questionnaire sustained injuries while on horseback, and only 18 of 29 (62%) of these patients were wearing helmets at the time of injury. The wearing of helmets while on horseback should be mandatory.

The use of headgear and other forms of protective equipment during equestrian activities has been extensively researched in the literature. Lim et al. reported an inverse relationship between compliance with helmets and the need for hospital admission [24]. The overall consensus is that compliance with the use of this equipment is low, with as few as 9% of individuals admitting to wearing helmets when their injury occurred [23]. The incidence of helmet use is higher in equestrians, particularly in a professional setting, due to regulations related to the sport [24]. In this work, responding to the questionnaire, 21 of 60 patients (35%) wore helmets at the time of their injuries. Of these, 62% wore helmets while on horseback and 10% while unmounted.

It is reasonable to assume that headgear usage or lack thereof may influence maxillofacial and non-maxillofacial injuries in this cohort. However, this study likely lacks the necessary power to show any significant difference in injury severity and need for surgery in those who did and did not wear helmets at the time of injury. However, conventional helmets do not provide adequate protection to the majority of the facial skeleton. This raises questions about the need for additional protective equipment or modification to currently available headgear.

Equestrian-related maxillofacial fractures are often associated with multiple concurrent non-maxillofacial injuries (15%). A report from the Irish National Spinal Injuries Unit over 10 years revealed that 40% of admissions were associated with equestrian activities [8]. The incidence of spinal trauma has been reportedly as high as 14% in some populations [6, 10, 25]. Fractures primarily affected the lumbar and thoracic spine and most commonly affected mounted riders. In this study, falls were shown to be significantly associated with a higher incidence of vertebral fractures, highlighting the importance of investigating for spinal trauma when an equine-associated facial fracture is recognised.

In addition, there is a disproportionate representation of traumatic head injuries in horse-related injuries. These injuries are a major contributor to mortality in this cohort [6]. In comparison to other sports, horse riding has the highest incidence of recreation-related traumatic brain injury [7]. A study by Abu Zidan et al. reported that one in five individuals with facial injuries had concurrent intracranial pathology, resulting in the death of two patients [26]. The risk of these injuries is higher in nonprofessional riders [27]. Our work demonstrated an association between frontal bone fractures and the incidence of head injury. Therefore, this mechanism should alert emergency physicians to the potential for serious injury. The incidence of head injury in this study (5%) appeared to be lower than previous reports in the literature. This is likely due to the direct referral of patients with equestrian-related head injuries to the National Neurosurgical Unit.

The association between the level of experience of riders and the risk of injury in equine sport has been investigated in previous publications. A study by Mayberry and colleagues found that amateur riders had a threefold increased incidence of injury compared with intermediate riders. This was eightfold higher compared to professionals [28]. These findings are supported by the work of other authors [29, 30]. It is worthwhile to note that in this current work, of those who responded to the questionnaire, there were only two professional national hunt jockeys (from a total of 29 mounted riders) who sustained maxillofacial injuries in comparison to 12 amateur riders (Table 3), supporting the information from previous studies.

The results of this study suggest that equipment to protect the face should be worn at all times when handling horses, particularly when unmounted. Published studies have shown that although a substantial proportion of horse-related maxillofacial injuries are sustained by unmounted riders, as seen in this study, helmets are rarely used or encouraged in this subgroup [16]. In addition to the suggestion to wear protective helmets at all times when handling horses, helmets should be designed to protect the middle and lower facial skeleton. It may be possible to improve helmet designs and certification tests to reduce the risk of facial injury in low-severity impacts and reduce the number of facial lacerations and nondisplaced fractures. The number of dentoalveolar fractures in this study is small (3 of 73 patients). This is likely due to the fact that dentoalveolar fractures in this setting are likely treated directly in local dental services. Nonetheless, the use of mouthguards should be promoted to offer protection against dentoalveolar injury.

To mitigate the risk of equine-related maxillofacial trauma, there is a need for a change in regulation and public education. Many injuries are avoidable, and public health efforts should focus on preventive measures, including education about horse behaviour, correct handling techniques, in addition to the use of appropriate safety equipment. It would also be prudent to promote the use of PPE in an occupational setting, given that the majority of maxillofacial injuries occur in unmounted handlers. These changes should be integrated with the implementation of safer riding practices and improved supervision, especially for amateur and juvenile riders.

The current work suffers from the inherent limitations of a retrospective study design. Rider experience was not documented for all individuals, which may have been a useful variable to correlate with injury. Finally, long-term follow-up is important to assess the impact of these maxillofacial injuries on return to work, sport, and quality of life.

The strengths of this research include its relatively large sample size, which includes 73 patients and 83 maxillofacial fractures, which is equal to or higher than previous work. The database from which the review was generated is prospectively maintained, ensuring a high level of accuracy. In addition, there was adequate documentation of concurrent head, spinal, and other body injuries.

## Conclusion

Horse-related maxillofacial injuries are not common, but may be severe. Kicks from horses were the most common mechanism of injury. There was no association between the mechanism of injury and injury pattern. Frontal bone fractures were associated with an increased likelihood of head injury and spinal trauma. Young males are the most commonly injured cohort, with the midface being the most common site of injury. Education should be promoted to increase the usage of helmets, whether mounted or unmounted, with particular emphasis on protecting the facial skeleton.
